# Conservative surgical approach towards placenta accreta spectrum disorders for uterine preservation

**DOI:** 10.1186/s12884-023-05370-6

**Published:** 2023-01-14

**Authors:** Shahul Hameed Mohamed Siraj, Kok Hian Tan, Ann M Wright

**Affiliations:** 1 Department of Minimally invasive Surgery Unit, Division of Obstetrics and Gynaecology, KK Women’s and Children’s Hospital, 100 Bukit Timah Road, Singapore, 229899 Singapore; 2 Department of Maternal Fetal Medicine, Division of Obstetrics and Gynaecology, KK Women’s and Children’s Hospital, 100 Bukit Timah Road, Singapore, 229899 Singapore; 3 OBGYN Academic Clinical Programme, DUKE-NUS Medical School, 8 College Road, Singapore, 169857 Singapore

**Keywords:** Caesarean section, Isthmocele, Residual myometrial thickness, Placenta accreta spectrum disorders

## Abstract

**Objective:**

We previously described a technique for repair of the myometrial defect at repeat Caesarean section which increases residual myometrial thickness thereby potentially reducing future niche-related complications. Here we describe how this technique can be modified for use for placenta accreta spectrum disorders, in line with emerging evidence that this is more a disorder of myometrial deficiency than morbid adherence.

**Design:**

The surgical performance of peripartum hysterectomy was compared with that of the modified technique in all women having repeat Caesarean delivery for placenta accreta spectrum disorder in a tertiary unit in Singapore between December 2019 and October 2021.

**Methods:**

Modification of the original technique involved the systematic delivery of the placenta starting from its most posterior attachment after uterine exteriorization. This is followed by the identification, mobilization, and apposition of the boundaries of myometrial defects as described previously.

**Results:**

Ten women had Caesarean hysterectomy and ten had Caesarean section using the modified approach. Age and gestational age at delivery were similar for the two groups. Women in the modified technique group had had fewer prior Caesarean sections and had a lower body mass index. Operating time, estimated blood loss and need for transfusion were all lower in the myometrial repair group but without statistical significance. There were no visceral injuries in the repair group but there was one bladder injury in the hysterectomy group.

**Conclusion:**

The modified approach provides an effective alternative to peripartum hysterectomy with favourable surgical profile and allows uterine conservation with restoration of myometrial thickness.

## Background

Although the lower uterine segment usually heals well after Caesarean section (CS), any loss of muscle during the repair, which can be substantial even after one CS, leads to niche formation [1]. Niche formation has been linked to several modifiable surgical factors including single layer closure and use of locking sutures. Both niche prevalence and depth increase with number of CS, prevalence rising from 62% after one to 78% after three CS depending on method of evaluation [2]. The nearer the scar is to the level of the internal os the higher the likelihood of a niche and the larger the defect [2]. Niche development may be accompanied by formation of new vascular connections within the scar area, which along with in-migration of a variety of inflammatory cells contributes to healing [3].

During future pregnancies the niche has the potential to become an isthmocele leading to complications including uterine rupture and placenta accreta spectrum disorders (PASD). As the niche deepens, the residual myometrial thickness (RMT) gets less, with the RMT further thinning during pregnancy as the niche widens. The longer and deeper the initial scar area, the larger the decrease in RMT seen in pregnancy and the larger the resultant defect [4]. The likelihood of developing complications in future pregnancy is related to the RMT, as it reflects the amount of muscle remaining. An RMT of less than 2 mm carries a significant risk of scar rupture [5].

The frequency of PASD has markedly increased over the last few years because of the increased Caesarean section rate. The risk is higher when placenta praevia complicates a pregnancy following previous CS, especially if the primary indication mandated a higher uterine scar and rises with each subsequent CS [6]. Historically treatment options centered round intentional retention of the placenta (IRP) with or without hysterectomy but latterly several techniques have been developed aimed at conserving the uterus including the Triple P [7] and Naussica [8] procedures.

Recent evidence of PASD being a disorder of defective decidua and uterine dehiscence rather than destructive trophoblastic invasion [9] suggests that a conservative approach with effective manual removal of placenta, when combined with peri-operative prophylactic haemostatic measures can be an optimal approach.

We have previously described a technique (Siraj et al.) for repair of the myometrial defect or isthmocele at the site of the previous scar at the time of repeat Caesarean Sect. [10]. The repair increases the RMT and reinforces the posterior uterine wall which is often thin in these cases. We now describe how this technique can be extended for use in cases of suspected PASD with focus on control of the major hemorrhage, in part due to the neovascularization around the scar which can be offered as an alternative to peripartum hysterectomy in women who request uterine conservation. This approach has the added advantage of offering opportunity to reconstitute the uterine wall by contemporaneous correction of the niche, potentially allowing future pregnancy and avoiding other niche-related complications.

## Methods

Our conservative surgical approach comprises the following:

### Preoperative assessment and peri-operative prophylactic haemostatic measures

For antenatally diagnosed cases of PASD the surgery is pre-planned and done by a dedicated team. The patient is counseled on surgical options and associated risks, an anesthetic review is performed, and insertion of internal iliac artery catheters (IIAC) is offered prophylactically to reduce peri-operative blood loss and blood is available. IIAC is a helpful but not essential part of the technique. For patients presenting as an emergency, IIAC insertion was only offered it time permitted. For an on-table diagnosis, IIAC insertion was not used.

Management depends on patient preference towards a conservative approach and needs to be established pre-operatively as the surgery is done under general anesthesia (GA). The method presented here is suitable for women wishing to avoid the prolonged follow up and potential risks associated with IRP but who wish for uterine preservation. Consent needs to include bleeding risk potentially necessitating hysterectomy, possible visceral injury, and potential for PASD recurrence or future scar pregnancy if uterine conservation is successful without ligation.

### Incision

Sites of both abdominal and uterine incisions need careful consideration when performing any CS for suspected PASD. A longitudinal skin incision which can initially be placed sub umbilically has advantages. It aids access both for adhesiolysis especially if the uterus is adherent to the anterior abdominal wall and for peripartum hysterectomy if required. It can also protect against inadvertent bladder injury if the bladder is drawn up. Extension offers access to the upper uterus should a classical or fundal uterine incision be chosen for placental avoidance, which itself allows placental retention should there be no signs of separation and uterine preservation is requested. However, a longitudinal skin incision may be less cosmetically acceptable and associated with more postoperative morbidity than a transverse wound.

A classical uterine incision on the upper segment may obscure the degree of placental separation leading to delay in controlling blood loss and difficulty in gaining access to the retracted muscle to repair the defect obligating a second transverse incision to be made for visualization, potentially increasing the risk of uterine rupture in any subsequent pregnancy. A transverse incision in the lower segment or isthmocele almost inevitably disturbs the placenta which can initially cause heavy bleeding and rules out placental retention. However, the use of a single uterine transverse incision through the previous scar has the advantage of allowing the placenta to be delivered in a systematic way under direct vision from its attachment on the posterior uterine wall first followed by removal from the neovascularized anterior wall while tracing the boundary of the sheared posterior myometrial defect prior to repair. This moderates the initial high blood loss from the neovascularised isthmocele associated with the more orthodox anterior placental separation and helps in the management of the often unrecognized bleeding from the posterior myometrial defect and the bleeding from the anterior inferior muscle close to the level of the internal os prior to repair.

We advocate entry through a pre-existing skin incision, usually transverse and suprapubic followed by a transverse incision through the upper third of the isthmocele above the level of the uterovesical fold through which the fetus and placenta are delivered. This avoids unnecessary dissection of the bladder and risk of renal tract injury as well as disruption of troublesome bridging vessels which run over the isthmocele and in the bladder serosa but does have the drawback of the patient being subject to a higher peripartum blood loss.

### Delivery of baby

Once the uterus has been entered, delivery is conducted in the usual way but needs to be expeditious. After delivery of the baby, the IIA balloons are inflated, and the uterus is exteriorized. This improves visualization of the operative field and provides access to the posterior uterine wall aiding both placental detachment and identification of the retracted posterior retracted muscle. It also allows manual compression of the uterine arteries helping reduce the blood loss while the myometrial rings are clamped.

### Delivery of placenta

A low-lying placenta may be situated within the isthmocele or attached to the previous scar depending on its level (within or above the endocervical canal). If the placenta is not adherent, it will separate spontaneously with or without uterotonics. If it is adherent, manual removal will be required. Regardless of method of placental delivery, both leave a non-retractile isthmocele which may be bleeding profusely from the retracted muscle rings at its boundary and the numerous bridging vessels lying in the serosa. We recommend a posterior rather than an anterior starting approach for the manual removal of the placenta to reduce the risk of bleeding from the aberrant vessels present anteriorly where the tissue planes are obscured. This has the added advantages of reducing risk of bladder injury and helping in identification of the posterior myometrial defect as described in the original method.

**Fig. 1:**
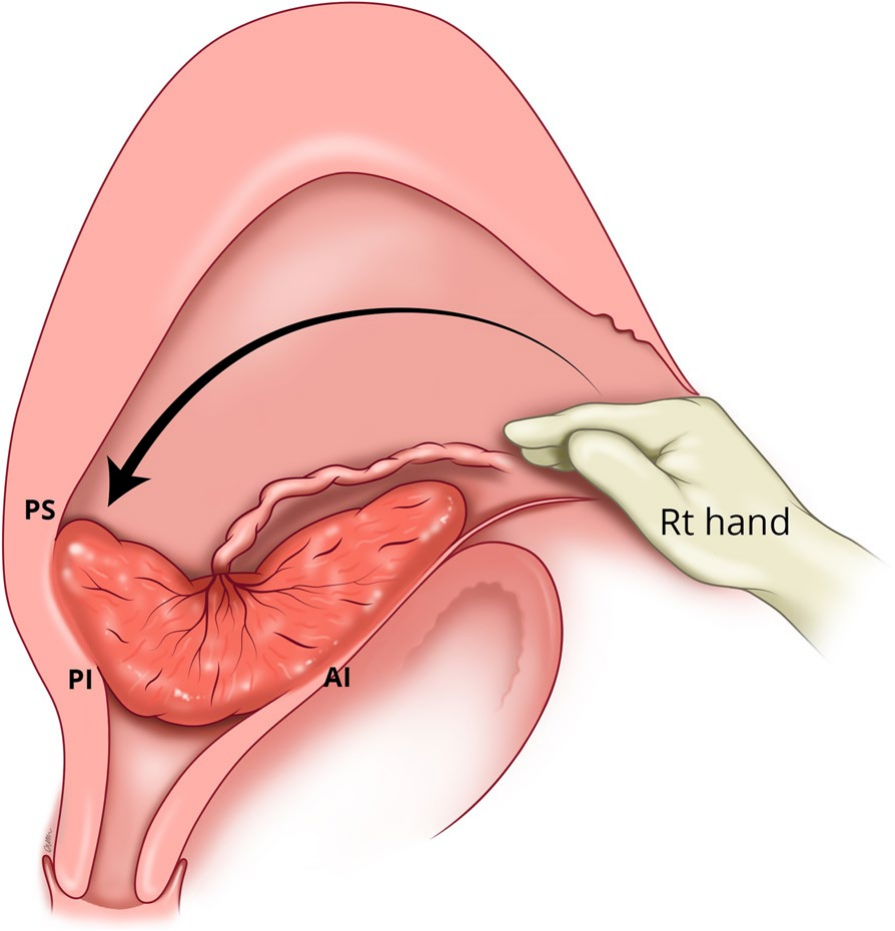
Hand through the incised isthmocele along the umbilical cord

**Fig. 2:**
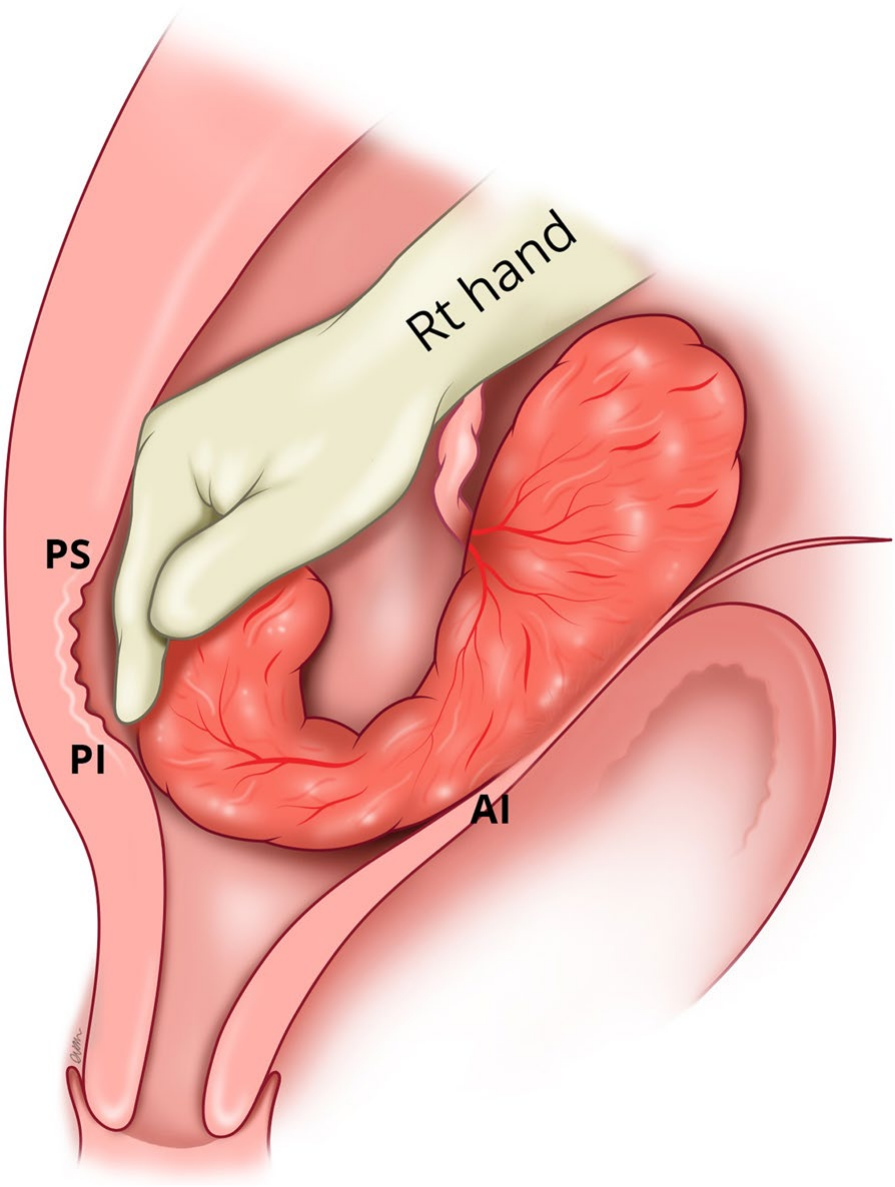
Separating the placenta posteriorly with hand and identifying the posterior defect of the uterus and the sheared boundaries of posterior superior (PS) and posterior inferior (PI) muscle layers

**Fig. 3:**
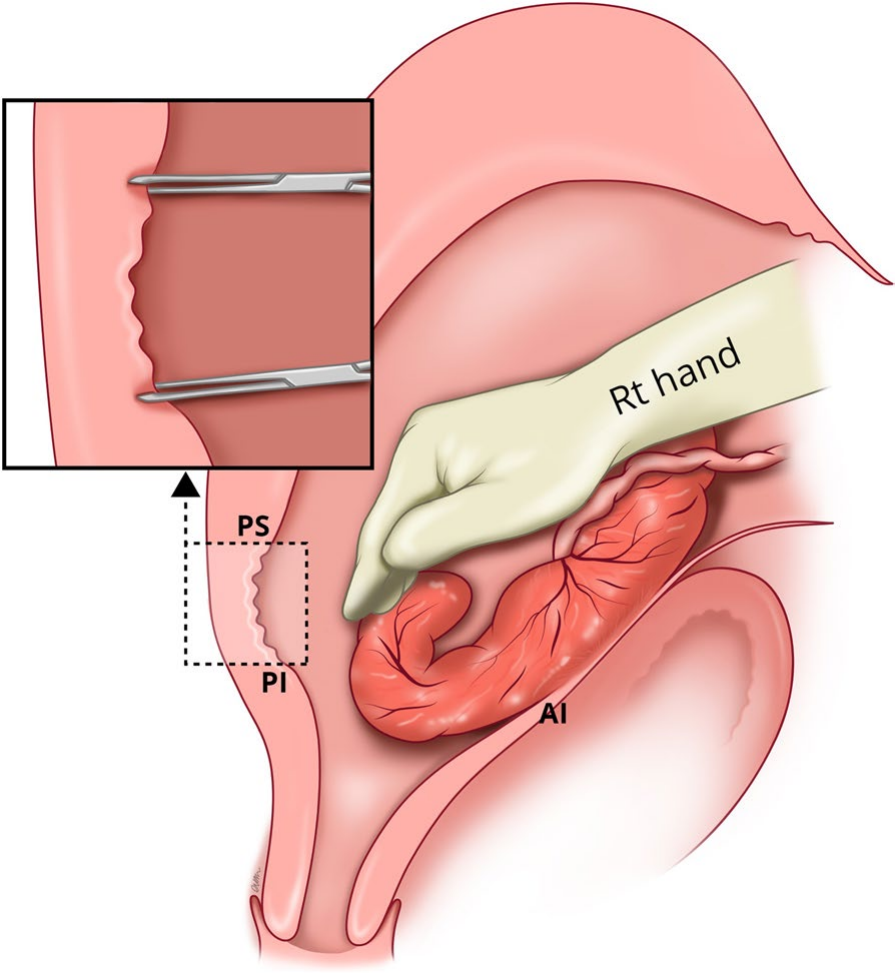
Application of the Green Armytage to posterior superior (PS) and posterior inferior (PI) muscle layers

**Fig. 4:**
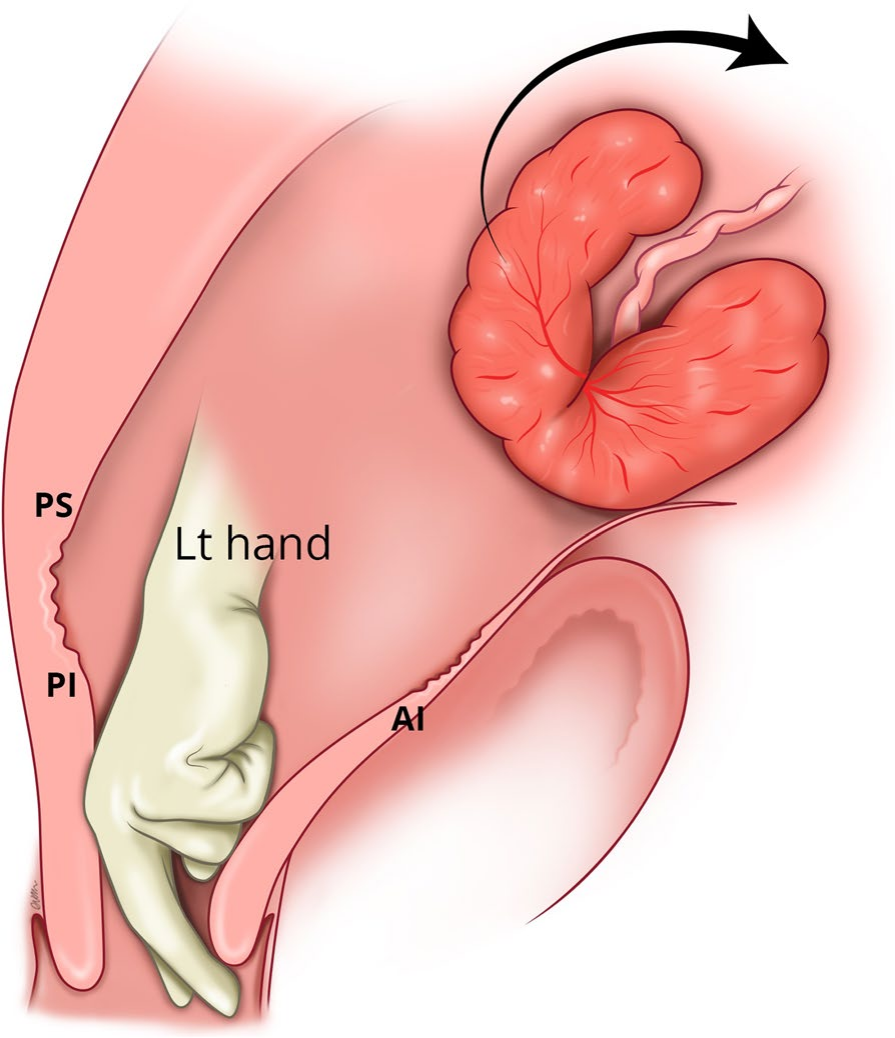
Insertion of the left-hand index /middle fingers through the cervix to reach the anterior fornix

**Fig. 5:**
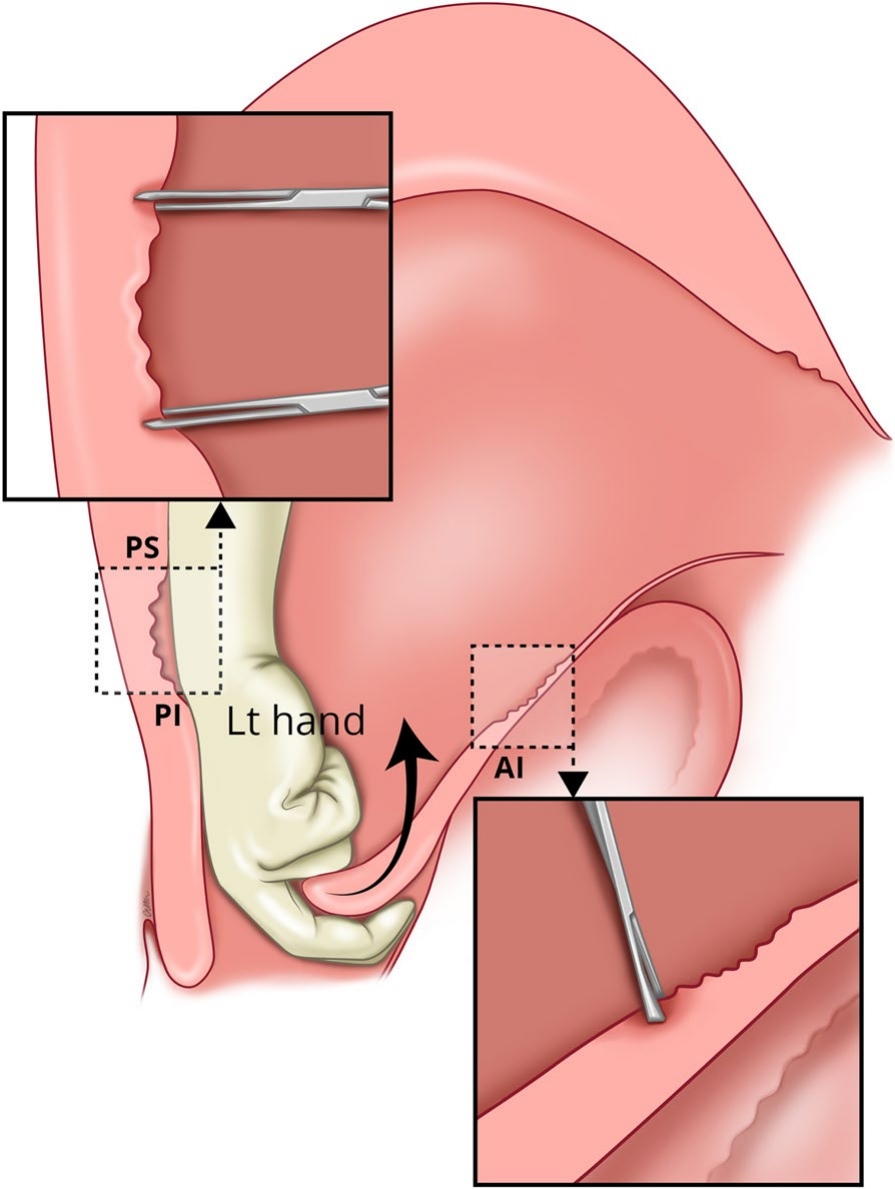
Lifting the cervix along the anterior fornix to facilitate identification and grasping of the anterior (AI) muscle at the base of the ischiocele, closer to the internal os, with the Green Armytage

**Fig. 6:**
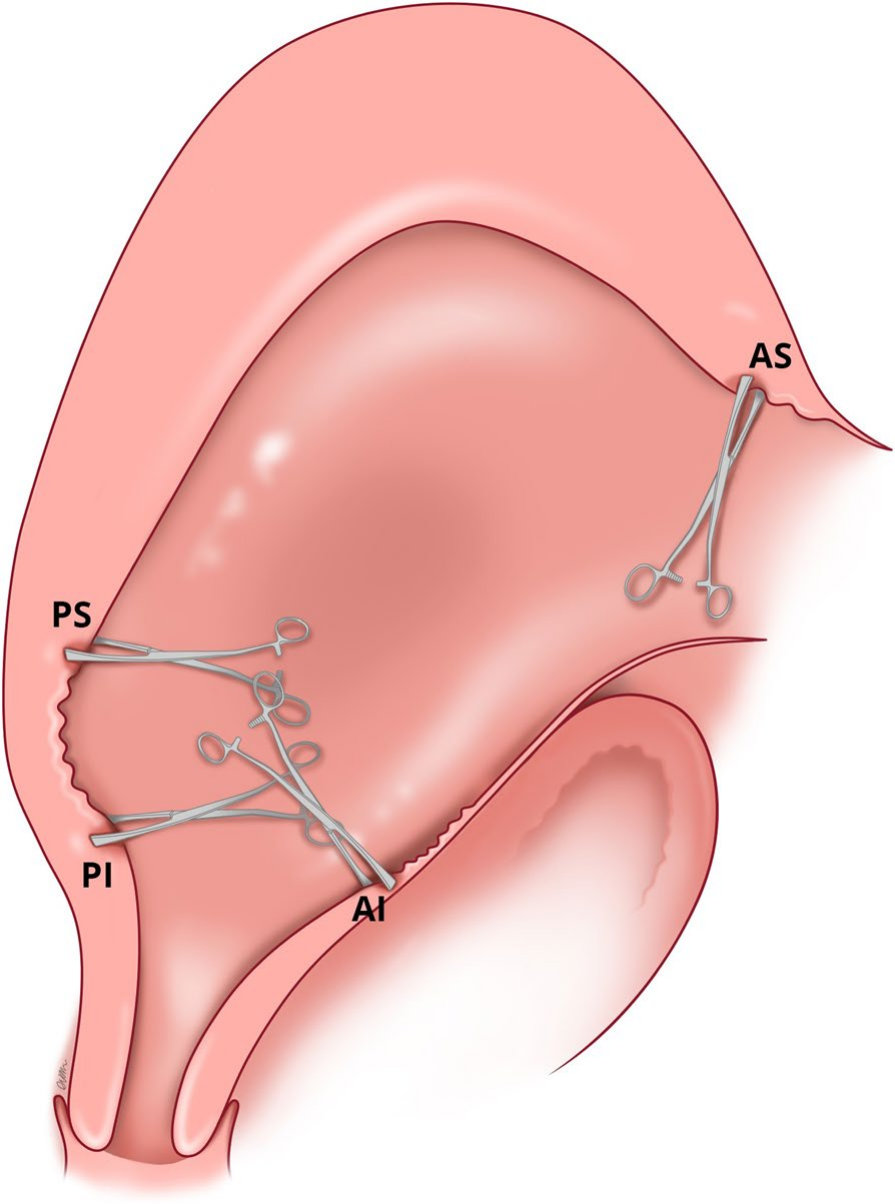
Application of Green Armytage including at the anterior superior muscle (AS) of the defect to facilitate repair of the anterior and posterior wall defects

If manual removal is required, we advocate the following steps:

1. After uterine entry the operator’s right hand is inserted through the incised isthmocele and directed towards the upper border of the most posterior aspect of placental attachment (Fig. 1) from where detachment is initiated, working laterally, bilaterally, to expose the posterior uterine wall defect (Fig. 2).
2. The exposed retracted inferior and superior posterior muscles are then grasped with Green Armytage clamps (Fig. 3). In some instances, the sheared posterior defect may be large and the inferior myometrial boundary difficult to identify. Recognition may be aided by the assistant’s right hand raising the outer aspect of the uterus below the utero sacral ligament facilitating application of the Green Armytage clamps to the lower posterior retracted muscle within.
3. Once the lateral aspects of the placenta are reached the operator’s left index and middle fingers are inserted into the endocervical canal (after digital dilatation, if necessary,) to the anterior fornix (Fig. 4) to lift the cervix (Fig. 5) and allow the retracted anterior inferior muscle edges to be identified and clamped, before detaching any residual attached placenta. This muscle ring is normally found at the level of the internal cervical os and needs to be isolated prior to application of clamps to avoid damage to the bladder base. Once the anterior ring of inferior muscle has been caught securely, the bleeding starts to slow, and the bladder can be separated safely from the lower segment of the uterus working from the lateral aspects medially. Pre-operative cleaning of the vagina can be performed during catheterization but with caution especially with major praevia.
4. The myometrial defect, the boundaries of which are shown in Fig. 6, is then repaired as previously described [10]. Success of this technique involves correct recognition of the retracted muscles at the boundary of the myometrial defect and repair of the muscle edges. As the posterior myometrial defect is closed and the uterine angles are secured the bleeding slows further allowing completion of the repair. Any redundant fascia forming the isthmocele, if not already ruptured, is incorporated into the anterior repair to build up the anterior uterine wall at the site of the scar and reduce the bleeding from the overlying aberrant vessels which can be difficult to control.

### Control of blood loss

Blood loss is at its highest immediately following placental delivery. This can be reduced by inflating the internal iliac balloons or applying a paracervical tourniquet, if feasible, while the edges of the myometrial defect are identified (Fig. 6) and secured. Uterine artery ligation can even be considered if necessary. Other measures which can be employed include the standard uterotonic drugs, balloon tamponade, blood transfusion, cell salvage and tranexamic acid.

We examined a consecutive series of twenty cases of PASD managed over a period of twenty-two months between December 2019 and October 2021. The study was conducted in a tertiary obstetric referral hospital in Singapore which has around 12,000 deliveries per year and a Caesarean section rate of 31–32%. The study was reviewed and approved by the SingHealth Centralised Institutional Review Board (IRB Approval Reference number – 2011/711/D). The surgical method adopted for each case was mainly governed by patient choice. A low-lying placenta was identified for all women on a mid-trimester pelvic ultrasound scan with characteristic ultrasonic features of PASD being identified on subsequent scans. Most of the patients also had an MRI. All deliveries were performed under GA and pre-operative intra iliac balloon catheterization was offered to all women having elective delivery and any being delivered as an emergency, if time permitted. We compared demographic data and peri-operative details as well as outcomes between groups. We looked at surgical time, estimated blood loss, need for transfusion, visceral injury, admission to the Intensive Care Unit and length of stay for our patients.

## Results

Of the twenty consecutive patients, ten had a Caesarean section with peripartum hysterectomy and ten were managed by Caesarean section using our technique. None of the woman had a Caesarean hysterectomy for failed conservative management. Five women in each group had the procedure performed electively between 34 and 36 weeks. The remainder needed emergency delivery for an intervening complication. All but one of the women who had had previous myomectomy and was delivered by CS with myometrial repair, had had at least one previous CS.

The groups were similar with respect to age and gestational age at delivery (Table 1). More women having hysterectomy had had two or more previous CS compared to women having CS and repair. Patients opted for hysterectomy had more caesarean section and it was their choices as family completed. The BMI was higher in the hysterectomy group although this did not reach statistical significance. The hysterectomy group had a longer surgical time (*p* = 0.05). Estimated blood loss (EBL) and need for transfusion were lower in the myometrial repair group but these did not reach statistical significance. There was one bladder injury in the hysterectomy group but no visceral injury in the conservative myometrial repair group.

**Table 1:** Comparision of patients demographic & perioperative details between peripartum hysterectomy and myometrial reapir

	**Peripartum Hysterectomy**	**Myometrial Repair**	***p* value**
**Number of Subjects**	10	10	
**Age (yrs) ± SD, Median**	35.1 ± 3.21, 34.5	34.4 ± 3.44, 33.5	0.644
**BMI ± SD, Median**	28.6 ± 4.70, 28.9	25.0 ± 4.90, 24.6	0.115
**Gestational age (wks)** ± **SD, Median**	34.0 ± 3.34, 34.9	34.5 ± 2.72, 34.5	0.695
**US**	10/10(100%)	10/10(100%)	1.00
Low lying placenta	2	2	
Features of PASD	6	6	
Placenta upper	1	0	
**MRI (%)**	4/10 (40%)	5/10 (50%)	
**Grading of PASD by MRI**	Accreta (2)	Accreta (2)	
	Increta (1)	Increta (2)	
	Percreta (1)	Percreta (1)	
**Grading of PASD by Histology**	Accreta (2)		
	Increta (4)	Not done	
	Percreta (2)		
**At least one features of PASD(US/MRI/Histology)**	8/10	9/10	
**Internal Iliac Artery Catheter (IIAC) (%)**	5/10 (50%)	5/10 (50%)	1.00
**Surgical time(min) ± SD, Median**	164 ± 62.3, 148	117 ± 27.1, 121	0.050
**Estimated blood loss(ml) ± SD** **(minimum – maximum)**	2570 ± 1739 (900–6000)	2090 ± 1385 (800–4000)	0.503
**Transfusion**	10/10 (100%)	8/10 (80%)	0.136
**Duration of hospital stay(day) ± SD, Median**	5.4 ± 2.2, 4.5	4.3 ± 1.2, 4.0	0.175
**ICU admission**	10/10 (100%)	8/10 (80%)	0.264
**Visceral injuries**	1 (bladder)	0	
**Previous number of CS**			0.096
1	3/10 (30%)	6/10 (60%)	
2	1/10 (10%)	3/10 (30%)	
3	4/10 (40%)	0/10 (0%)	
4	2/10 (20%)	1/10 (10%)	

## Discussion

### Main findings

All clinical options for PASD after delivery of the fetus carry potential complications including major hemorrhage. IRP can lead to sepsis and secondary hemorrhage requiring delayed hysterectomy. Hysterectomy results in permanent inability to have more children regardless of family planning wishes although even with uterine conserving techniques contemporaneous sterilization is often offered to avoid recurrence. Peripartum hysterectomy carries additional surgical morbidity related to the need to remove the cervix. It has been associated with a 7% ureteric injury and 15% rate of bladder injury [7].

The Triple-P procedure, which involves delivery of the fetus above the placenta and resection of the myometrium with attached placenta after pelvic devascularization, was found to have fewer bladder injuries and no ureteric injury compared with hysterectomy but was complicated by delayed primary hemorrhage requiring embolization or re-laparotomy for intra-abdominal bleeding from neovascularization of the bladder serosa [7]. Other conservative surgical techniques are time consuming and associated with high operative risk most notably bleeding and visceral damage.

Our conservative procedure, with favorable surgical outcomes, differs from the Triple-P and other procedures by focusing on formal recognition and repair of the pre-existing myometrial defect whose muscle edges are actively bleeding after delivery to restore tissue integrity. Blood loss is further controlled using several maneuvers including expeditious exteriorization of the uterus, applying manual compression at the level of the uterine arteries, and systematically delivering and detaching the placenta from posterior wall first and working anteriorly; all of which can be supported using IIA balloons and other measures to achieve haemostasis.

The technique, which focuses on conservation and restitution of the uterus rather than excision, provides an alternative to other conservative surgical approaches for placenta accreta spectrum disorder and has a comparable surgical profile. It has the added advantage of addressing the myometrial niche with implications for patient’s future symptomatology.

### Strengths and limitations

Our conservative surgical approach for placenta accreta spectrum disorder has a favourable surgical outcome but does depend on a full understanding of the pathophysiology of the placental condition and the steps required for optimal surgical correction of the myometrial deficiencies. The approach has the strength of conserving the uterus with adequate myometrium thickness, reducing the risks of gynaecological and obstetrical complications later.

The limitations relate to the need for a comprehensive multidisciplinary team with experience in addressing large blood loss, careful fluid management and anaesthetic input, a practice which may be difficult to achieve in a non-tertiary setting, especially if case numbers are small. In such a situation, a regional network could be beneficial. Our study is also a small consecutive series.

We acknowledge the inevitable selection bias in this study produced by the choice of surgical approach being made by the individual patient after objective discussion of their options. Although there may be some uncertainty of the PAS diagnosis for women who underwent myometrial repair and data for one woman in each group is incomplete, all the other women had at least one diagnostic information suggestive of PAS, either from ultrasound scan, MRI or histology strongly supportive of the diagnosis; and several had all three.

### Interpretation (in light of other evidence)

There has been recent evidence of PASD being a disorder of defective decidua and uterine dehiscence rather than destructive trophoblastic invasion [9]. When there is a high index of suspicion for PASD, there is often pre-operative uncertainty around the true extent of the problem due to lack of sensitivity of screening tools including obstetric ultrasound and pelvic MRI, for both detection and determination of extent of uterine wall involvement. In our experience, in patients with previous Caesarean section with PASD, a low-lying placenta in the current pregnancy is invariably found to be occupying the isthmocele, rather than to be adherent to it, consistent with PASD being a disorder of defective decidua and uterine dehiscence rather than a disease of trophoblastic invasion. The integrity of the myometrium in the area of placental attachment in the anterior wall, rather than the placentation itself, impedes safe access to avascular dissection planes. Thus, our starting approach from the posterior uterine wall for removal of placenta is aimed at improving the speed and safety of the procedure. Our results supported this approach. Future research, in the form of a larger prospective study, is required to further assess its morbidity, performance relative to other conservative surgeries and the subsequent reproductive and gynaecological outcomes.

## Conclusion

This surgical approach involving delivery of baby through the incised isthmocele, expeditious uterine exteriorization and systematic manual removal of the placenta from posterior uterine wall to anterior, combined with our previously described technique of myometrial defect repair, can optimally conserve the uterus while reducing the likelihood of future niche complications in cases of PASD. It is performed within the uterine boundary which reduces the risk of perioperative complications. As it is designed for use in high-risk situations, where alternatives are equally fraught with risk, including life threatening bleeding, training, practice, and experience in the technique is obligatory.

## Fundings

The study is supported by Singapore Duke-NUS Benjamin Henry Sheares Professorship in Obstetrics & Gynecology and Integrated Platform for Research in Advancing Metabolic Health Outcomes of Women & Children (IPRAMHO) Study Group (NMRC CGAug16C008). The funding was used to cover the costs of medical illustration and open access submission.

## Data Availability

The dataset generated and analysed in this study, is not available for the public. Data can be made available on request from researchers who meet the criteria for access to patient's confidential data and upon approval from the IRB. Data supporting the conclusions of the study is available with Dr. Shahul Hameed Mohamed SIRAJ, Department of Minimally Invasive Surgery, Division of Obstetrics and Gynecology, KK Women’s and Children’s Hospital, Singapore(drshmsiraj@yahoo.com).

## Abbreviations

PASD: Placenta accreta spectrum disorders
CS: Caesarean section
AS: Anterior superior muscle
AI: Anterior inferior muscle
PS: Posterior superior muscle
PI: Posterior superior muscle
